# Impact of COVID-19 on diagnosis and testing for TB in a high-resource, low-burden setting

**DOI:** 10.5588/ijtld.22.0132

**Published:** 2022-09-01

**Authors:** A. J. Jones, E. C. Jones-López, S. M. Butler-Wu, M. L. Wilson, J. Rodman, L. Flors, C. Voyageur, B. E. Jones

**Affiliations:** 1 Martin Luther King Jr. Center for Public Health, Los Angeles, CA, USA; 2 Keck School of Medicine, University of Southern California, Los Angeles, CA, USA; 3 Los Angeles County and University of Southern California (LAC +USC) Medical Center, Los Angeles, CA, USA

Dear Editor,

The COVID-19 pandemic has negatively impacted TB control globally.^1^–^9^ Throughout the United States, there has been a downward trend in the number of reported cases of TB disease.^10^ In 2020, there were 19.4% fewer reported cases (7,174, rate of 2.2 per 100,000) compared to 2019 (8,920, rate of 2.7 per 100,000). In 2019, the state of California had the highest number of cases of TB disease (2,112, rate of 5.4 per 100,000), which declined in 2020 (1,705, rate of 4.3 per 100,000).^11^ This 19.3% decrease in TB disease incidence in California during 2020 is similar to the decrease in the total incidence of TB disease in the United States (19.4%).

Following the Correspondence by Louie et al,^12^ which reported a 60% decrease in medical evaluations for signs and symptoms of TB from February 2020–May 2020 in the San Francisco Department of Health TB Clinic, we reviewed diagnosis and testing for pulmonary TB disease during 2019 (pre COVID-19 period) and 2020 (COVID-19 period). The study was conducted at the largest public hospital in Los Angeles County (LAC) and the University of Southern California Medical Center (USC Medical Center). During the study period, LAC reported >700,000 SARS-CoV-2 infections,^13^ placing LAC at the juxta-position of COVID-19 and TB. The first patient with COVID-19 at LAC+USC Medical Center was diagnosed in March 2020, and shortly thereafter in April, we diagnosed our first case of pulmonary TB and COVID-19 co-infection.

Our primary aim was to evaluate the impact of COVID-19 on pulmonary TB diagnosis by reviewing whether there would be a meaningful change in the frequency of sputum collection for acid-fast bacilli (AFB) and/or AFB smear positivity during the COVID-19 pandemic (2020) compared to the prior period (2019). We hypothesized that there would be a decrease in TB diagnosis during the COVID-19 period, which could be secondary to either a decrease in providers ordering TB tests, or a delay in patient presentation for care due to stay at home ordinance and attempts to maintain social distancing. We reasoned that a delay in patient presentation would lead to a shift towards more severe TB disease at diagnosis, with a higher proportion and grading of AFB smear positivity and/or more advanced disease on chest radiograph (CXR). This is a non-interventional retrospective study for which we reviewed microbiology data, as well as CXR and computed tomography (CT) scans for patients diagnosed with pulmonary TB (≥1 respiratory sample culture positive for *Mycobacterium tuberculosis*) during the period January 1, 2019 to December 31, 2020. Chest imaging studies were independently reviewed by a radiologist (LF). Disease severity on chest imaging was determined by the extent of the thoracic cavity involvement; with less than 25%, 25–50%, and >50% of thoracic cavity involvement, which corresponded to mild, moderate, and extensive disease, respectively.^14^ At our hospital, tests for pulmonary TB include three sputum samples for AFB smears and AFB cultures; two sputum samples for Xpert^®^ MTB/RIF (Cepheid, Sunnyvale, CA, USA); and CXR. Chest CT scans were also available for 23/41 (56%) patients diagnosed with *M. tuberculosis* culture-positive pulmonary TB during 2019 and for 18/28 (64%) TB patients during 2020. Xpert is routinely performed on all first and second sputum samples received for AFB culture. In contrast, there is no maximum number of AFB cultures which can be performed. Our primary outcomes were to measure: 1) the number of pulmonary TB tests (sputum samples for AFB smears and cultures) per year, 2) the number of culture-positive pulmonary TB diagnoses per year, 3) sputum AFB smear microscopy positivity rate per year, and 4) severity grading of chest imaging studies. Using a two-sample test for proportions, we analyzed the distribution of these four primary outcomes in two time periods, pre-COVID-19 (January 1, 2019–December 31, 2019) and then during the COVID-19 pandemic (January 1, 2020–December 31, 2020).

A total of 4,437 sputum samples for AFB smears and cultures were included in the analysis. We found that the total number of sputum AFB smear and culture samples collected decreased 37% during the COVID-19 period (*n* = 1,712) compared to pre-COVID-19 (*n* = 2,725) (Table). The absolute number of patients diagnosed with pulmonary TB in 2020 decreased from 41 to 28. However, despite the decrease in absolute number of pulmonary TB diagnoses and TB tests, the proportion of positive *M. tuberculosis* cultures was similar for the two periods (1.5% pre-COVID-19 vs. 1.6% during COVID-19; *P* = 0.45). CXR evaluation for moderate and extensive TB disease did show statistical significance as a measure of disease severity, which suggests patients had more extensive TB disease at the time of diagnosis during the COVID-19 study period (Table). Several other measures of disease severity, including AFB smear positivity and cavitary lesions on CXR and chest CT scans, were measurably higher, but not statistically significant.

**Table:** Summary of results of patients diagnosed with pulmonary TB from January 1, 2019 to December 31, 2020 at LAC+USC Medical Center.

Variable (per patient)	2019 *n* (%)	2020 *n* (%)	*P* value
Total sputum samples collected for AFB smears/culture, *n*	2,725	1,712	—
Sputum *M. tuberculosis* culture-positive	41 (1.5)	28 (1.6)	0.732
Sputum AFB smear-positive	24/41 (58.5)	17/28 (60.7)	0.857
Moderate or extensive disease (CXR)	18/41(43.9)	19/28 (67.8)	0.050^*^
Cavitary lesion (CXR)	21/41 (51.2)	17/28 (60.7)	0.436
Moderate or extensive disease (chest CT)	17/23 (73.9)	12/18 (66.7)	0.613
Cavitary lesion (chest CT)	9/23 (39.1)	11/18 (61.1)	0.162

* Significant at *P* = 0.05 (two-sample test for proportions).

LAC +USC = Los Angeles County and University of Southern California; AFB = acid-fast bacilli; CXR = chest X-ray; CT = computed tomography.

This study has some limitations. Although our sample size was large, the number of patients diagnosed with pulmonary TB was relatively small, which may have impacted our conclusions. Additionally, the time periods used in this study may have led to misclassification: we started the 2020 analysis in January as opposed to March when COVID-19 cases were first detected, so may have attributed some of the pre-COVID-19 cases to the COVID-19 period. Finally, we did not include clinical variables, which may have been helpful in interpreting the results.

In conclusion, we found a 37% decrease in TB tests (sputum samples sent for AFB smears and cultures) during COVID-19 (2020) compared to the period immediately prior (2019). The decrease in number of reported cases of TB raises concerns for potentially undiagnosed TB in a community where approximately 80% of TB disease results from the reactivation of TB infection. Although the proportion of patients diagnosed with pulmonary TB did not change, chest imaging indicated patients who presented in 2020 had more severe disease. These patients could have delayed seeking medical care during the pandemic leading to more severe disease state on presentation. These patients would therefore be infectious for longer, with increased possibility of transmitting TB to close contacts prior to diagnosis. In this relatively small study, the differences in the other measured variables are not significant, but at a country-wide level these relatively small differences may have a large impact on public health.
